# Molecular diagnostic in fetuses with isolated congenital anomalies of the kidney and urinary tract by whole‐exome sequencing

**DOI:** 10.1002/jcla.23480

**Published:** 2020-08-11

**Authors:** Xiaoyan Zhou, Yan Wang, Binbin Shao, Chen Wang, Ping Hu, Fengchang Qiao, Zhengfeng Xu

**Affiliations:** ^1^ State Key Laboratory of Reproductive Medicine Department of Prenatal Diagnosis Women's Hospital of Nanjing Medical University Nanjing China; ^2^ Department of Obstetrics The Affiliated Huai an No. 1 People's Hospital of Nanjing Medical University Huai an China

**Keywords:** clinical impact, isolated CAKUT, perinatal management, whole‐exome sequencing

## Abstract

**Background:**

In prenatal care, accumulating evidences has demonstrated that whole‐exome sequencing (WES) expedites the genetic diagnosis of fetal structural anomalies. However, the clinical value of WES in the diagnosis of prenatal isolated congenital anomalies of the kidney and urinary tract (CAKUT) is unknown.

**Methods:**

Forty‐one fetuses with unexplained isolated CAKUT, normal karyotype and negative chromosomal microarray analysis (CMA) results, underwent WES and were accordingly grouped as (a) Group 1: complex cases with bilateral renal abnormalities (N = 19); and (b) Group 2: cases with isolated unilateral fetal renal abnormalities (N = 22).

**Results:**

The detection rate of WES for pathogenic variants and incidental variants was 7.32% (3/41) and 2.4% (1/41), respectively. The three pathogenic variants were identified in the genes *ACTA2* (multisystem smooth muscle dysfunction syndrome), *PKHD1* (autosomal recessive form of polycystic kidney disease), and *PKD1* (autosomal dominant polycystic kidney disease type 1). The incidental variants were detected in genes *PPM1D* (syndromic neurodevelopmental disorders). Furthermore, all above fetuses carrying pathogenic variants came from bilateral kidney anomalies. Thus, the detection rate was 0 for fetuses with unilateral fetal renal *abnormalities* and 15.7% (3/19) for bilateral renal abnormalities.

**Conclusion:**

This cohort shows that prenatal WES is a supplementary approach for the etiologic diagnosis of unexplained isolated CAKUT with negative CMA, especially for fetuses with bilateral renal abnormality.

## Graphical abstract

Flowchart of characteristic of pregnant women and diagnostic finding from WES (analysis of fetuses with isolated CAKUT). Forty‐one cases with isolated CAKUT and prenatal diagnosis results available (WES) were selected. Detail results are described in the main text. CAKUT: congenital anomalies of the kidney and urinary tract WES: whole‐exome sequencing.

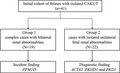

## 1 INTRODUCTION

Congenital anomalies of the kidney and urinary tract (CAKUT), the incidence of which is around 0.2% by prenatal sonography, have been reported to accounts for 40%‐50% of the global pediatric end‐stage renal disease (ESRD) as well as 7% of global adult ESRD.^1^ Accumulating evidences from animal and human studies suggest that genetic factors contribute to CAKUT.^2^ To date, more than 200 clinical syndromes were described phenotypes of CAKUT,^3^ and approximately 40 monogenic genes were identified to cause renal and urinary abnormality if mutated, which explained 5%–20% of CAKUT cases.^4^ In prenatal care, microarray approaches are currently most recommended as the first‐line genetic test for fetuses with CAKUT, achieving over 3.8% incremental yield of detecting pathogenic copy number variants (CNVs) in cases with normal karyotype.^5^ However, most definitive diagnoses are challenging as part of the monogenic origin and the phenotype heterogeneity during an ongoing pregnancy.^6^

Whole‐exome sequencing (WES) has been widely applied as a first‐line clinical diagnosis, especially in children's metabolic and neurodevelopmental disorders. In prenatal care, two large‐scale prenatal studies suggested that WES facilitates the diagnosis of fetal structural anomalies, enabling fetal prognosis with higher accuracy while reducing the risk of recurrence in future pregnancies.^7^, ^8^ However, previous studies have reported many challenges regarding the clinical application of WES in prenatal care, besides prenatal phenotyping limitations and technical problems, the counseling was also challenged with incidental or secondary findings, as well as variants of uncertain significance.^9^, ^10^ Meanwhile, it was suggested that diagnostic yields were higher in multiple fetal anomalies.^10^ However, the observations of most CAKUT by prenatal sonography were isolated and sporadic, which suggests that genetic origin that potentially contributes to this cohort should be considered.^11^ In the field of prenatal study, the utility of WES for the diagnosis of fetal CAKUT remains uncertain and the effectiveness and feasibility of WES in prenatal urinary malformation, especially isolated sporadic cohort, needs to be further investigated.

In the present study, we performed WES analysis upon 41 isolated sporadic CAKUT cases with negative CMA results, exploring the application value of WES in the perinatal management of the cohort by means of evaluating the phenotypic, genotypic, and prognosis outcomes.

## 2 METHODS

### 2.1 Participant recruitment and sample collection

This is an observational cohort study of pregnant women attending the Nanjing Maternity and Child Health Care Hospital (Jiangsu, China) for prenatal diagnosis from January 1, 2012, to June 1, 2018. This study was approved by the Medical Ethics Committee of Nanjing Maternity and Child Health Care Hospital. All data were prospectively collected from our patient files. The inclusion criteria for fetuses were isolated renal abnormalities detected by the 22‐24 weeks anomaly screening as prenatal ultrasound showed no signs of other fetal structural anomalies. Informed consent was obtained from parents. For fetuses whose tests revealed aneuploidy or CNVs which justified the fetal anomalous phenotypes, they were excluded from subsequent analyses. Then, a total of 41 subjects with negative CMA results (CMA results presenting no pathogenic genetic cause) were subsequently subjected to WES, including 19 cases with bilateral renal abnormalities and 22 cases with unilateral renal abnormality (URA), while unilateral kidney anomalies in combination with oligohydramnios which suggested possible kidney affection were also considered to be bilateral kidney anomalies. The inclusion criteria for URA were unilateral renal agenesis, multicystic dysplastic kidney, or kidney hypoplasia. We made clear and professional communications with parents about the expectations and worries, thus ensuring the parents with a thorough understanding before taking part into such testing. Participants were informed that only CAKUT‐related pathogenic or likely pathogenic results detected by the ultrasound scan were to be returned. Incidental findings which were not related to the primary test indication and the variants located in genes included in the American College of Medical Genetics and Genomics (ACMG) secondary findings list were not evaluated or reported if not requested.^12^

### 2.2 Extraction of genomic DNA

Invasive sampling was carried out by amniocentesis. Genomic DNA of the fetuses and parents was, respectively, collected from amniotic fluid and peripheral blood by routine methods. A Qubit Fluorometer (Thermo Fisher Scientific) and electrophoresis were utilized to conduct the quality and quantity control of genomic DNA.

### 2.3 Whole‐exome sequencing

Five hundred nanograms of genomic DNA from the fetuses and their parents (if available.) were used for WES. Sonication and target enrichment prepared sheared samples for fragment libraries establishment by utilized of a AgilentSureSelect QXT ALL Human Exon V6 kit according to the manufacturer's instructions. The captured DNA was amplified by solid‐phase bridge PCR and subsequently paired‐end sequenced on Illumina Hiseq 2500 (Illumina,Inc). Alignment of reads to the human reference sequence (hg19 assembly) and variant detection was conducted with Genome Analysis Toolkit 3.4 (GATK, www.broadinstitute.org/gatk). The annotation information of variations was obtained from seattleseq Annotation. Variants were filtered against 1000 Genomes, the dbSNP database, the Genome Aggregation Database, the Online Mendelian Inheritance in Man database, and the Human Gene Mutation Database. All experimental and analytical methods were performed according to the previous reports.^13^

### 2.4 Variant validation by PCR and Sanger sequencing

The pathogenic and likely pathogenic variations in fetal phenotype‐related genes were validated by Sanger sequencing. All primer designs were generated with the Primer Premier 5 software (Premier Biosoft International, PCR conditions available on request). Sanger sequencing was completed on the ABI 3730xl DNA analyzer (Applied Biosystems).

### 2.5 Variation analysis

All candidate variants were defined as pathogenic, likely pathogenic, of uncertain significance, likely benign or benign following the instructions of the ACMG guidelines,^14^ and the results were subsequently reviewed by pediatric nephrologist, clinical geneticist, and pediatric radiologist, respectively. The pathogenic and likely pathogenic variants in fetal phenotype‐related genes were reported to the parents. Meanwhile, the secondary findings and the incidental findings which were unrelated to the fetal CAKUT phenotypes but known to cause moderate to severe childhood onset disorders were only reported when parents requested for the results.

## 3 RESULTS

### 3.1 Demographic characteristics and pregnancy outcome

The median age of pregnant women in this study was 31 years old, and the gestational age (GA) varied between 22 and 32 weeks (median GA = 23 weeks). The fetuses were from healthy non‐consanguineous parents. All cases with no family history of CAKUT, only one case whose mother had congenital left kidney agenesis. Of the 41 fetuses, 19 exhibited bilateral prenatal kidney anomalies, including bilateral enlarged or hyperechogenic kidneys, bilateral kidney hypoplasia and bilateral multicystic dysplastic kidney (MCDK) et al Detailed information about prenatal findings in each fetus is presented in Table 1. 89% (17/19) of bilateral cases were combined with oligohydramnion and underwent termination of pregnancy (TOP). Additionally, two cases were delivered at full term, which were diagnosed with bilateral enlarged or hyperechogenic kidneys by prenatal ultrasound. Accordingly, the postnatal ultrasound detected kidneys abnormality including 1 case of left polycystic kidney disease and 1 case of diffuse renal disease. No developmental abnormalities were detected in the follow‐up interviews.

**Table 1:** Prenatal ultrasound diagnoses in fetuses included in the study

Prenatal kidney diagnoses	Number of cases
Bilateral renal abnormalities	19
Bilateral hyperechogenic kidney, bilateral hydronephrosis and megalo‐ureter, megabladder	1
Bilateral enlarged or hyperechogenic kidneys	7
Bilateral hyperechogenic kidneys, megabladder	1
Bilateral kidney hypoplasia/ bilateral MCDK	5
Right kidney dysplastic? Left renal agenesis?	1
Right renal agenesis, left megalo‐ureter	1
Left hyperechogenic kidney, right multicystic dysplastic kidney	1
Right renal agenesis, left renal dysplasia	1
Left hyperechogenic kidney? right renal agenesis?	1
Unilateral MCDK/kidney dysplasia/adysplasia/cystic kidney/renal agenesis	22
Total number of fetuses	41

Abbreviations: MCDK, multicystic dysplastic kidney.

### 3.2 Pathogenic variations

DNA samples from 41 fetuses without causal anomalies on CMA were detected by WES. Pathogenic or likely pathogenic variants in *PKD1, ACTA2*, and *PKHD1* genes were detected, and the diagnostic rate of WES was 3/41 (7.3%) (Table 2). Moreover, the above‐mentioned genes were all identified in cases showing bilateral hyperechogenic kidneys combined with other anomality: Case 1 with enlarged kidneys; Case 2 with bilateral hydronephrosis, megalo‐ureter, megabladder, and oligohydramnion; and Case 3 with enlarged kidneys and oligohydramnion.

**Table 2:** Phenotype and genotype information for the study

Case	Prenatal ultrasound findings	Gene	Alteration	Classification	Diagnosis syndrome	Classification of finding	Origin	Postnatal ultrasound findings
1	Bilateral hyperechogenic enlarge kidneys	PKD1	c.6571C > T p.Arg2191Cys	Likely pathogenic	ADPKD	Diagnostic finding	De novo	TOP
2	Oligohydramnion,bilateral echogenic kidneys, bilateral hydronephrosis,megalo‐ureter,megabladder	ACTA2	c.536G > A: pR179H	Pathogenic	Multisystemic smoth muscle dysfunction syndrome	Diagnostic finding	De novo	TOP
3	Oligohydramnion, bilateral hyperechogenic and enlarge kidneys	PKHD1	c.8301del p.N2768fs*18	Pathogenic	Polycystic kidney disease 4,with or without hepatic disease	Diagnostic finding	*Inherited*	TOP
			c.4481del p:N14947fs*6	Pathogenic	Polycystic kidney disease 4,with or without hepatic disease	Diagnostic finding	*Inherited*	TOP
4	Right renal dysplasia	PPM1D	c.1434delC p.R2191C	Pathogenic	Neurodevelopmental disorders syndrome	Incidental finding	De novo	Right renal

Abbreviations: ADPKD, autosomal dominant polycystic kidney disease; TOP, (late) termination of pregnancy.

In Case 1 which was diagnosed with bilateral echogenic kidneys by ultrasound examination at 26 weeks' gestation, a likely pathogenic variant, c.6571C > T, in a CAKUT‐associated gene *PKD1* was detected by WES. The de novo homogeneous c.6571C > T mutation, which resulted in a replacement of arginine (Arg) in position 2191 with cysteine (Cys) (p.Arg2191Cys), was defined as “likely pathogenic” (PS2 + PM1 + PM2 + PP3 + PP4) according to the ACMG guidelines. *PKD1* gene is involved in the development of renal tubular, and its mutations were reported to induce autosomal dominant polycystic kidney disease type 1 (ADPKD1). Generally, pathogenic variants in *PKD1* are phenotypically severe with higher incidence of ESRD and arterial hypertension.^15^ Case 2 involved several ultrasound abnormalities including oligohydramnion, bilateral echogenic kidneys, bilateral hydronephrosis, megalo‐ureter, and megabladder and was detected of a de novo nonsynonymous SNV (c.536G > A; p.R179H) in exon6 of *ACTA2* gene. Mutations in *ACTA2* gene was demonstrated to induce smooth muscle cells (SMC) disruption, while the contraction and relaxation of SMCs are crucial for the normal operations of blood vessels, as well as the digestive, respiratory, or urogenital system. Moreover, p.R179H mutation in *ACTA2* gene is the main cause for multisystemic smooth muscle dysfunction syndrome (MSMDS) and accordingly facilitates in genetic counseling and reduces reproductive hazards. The pregnancy of Case 2 was terminated due to the genetic disorder at the 25th week. In Case 3 at 22 weeks' GA, the ultrasound abnormalities were bilateral hyperechogenic and enlarged kidneys combined with oligohydramnios. WES detected two frame shift deletion (c.8301del:pN2768Tfs*18 and c.4481del:p.N1494Tfs*6) in the *PKHD1* gene. These variants were defined as “pathogenic” according to the ACMG guidelines. *PKHD1* participates in the cellular division process of centrosome duplication and mitotic spindle assembly, and its dysfunction could induce mitotic defects, which contribute to cystogenesis in autosomal recessive form of polycystic kidney disease (ARPKD).^16^ The pregnancy of Case 3 was terminated due to oligohydramnios at the 27th week.

### 3.3 Incidental findings

We detected incidental findings in Case 4 with unilateral renal agenesis (Family 38) and a de novo frame shift variant (c.1434delC) in *PPM1D*, suggesting a loss‐of‐function mechanism (Table 2). This variant was defined as “pathogenic” according to the ACMG guidelines. Previous reports demonstrated the presence of *PPM1D* gene in fetal and adult human brain tissues,^17^ which is integrally involved in the mild to severe intellectual disability (ID), developmental delay, behavioral problems such as anxiety disorders, hypotonia, and periods of fever and vomiting,^18^ while no renal phenotype was described. Since the above‐mentioned features were not compatible with the prenatal findings in our study, we therefore defined the variant as an incidental finding. In respect to the indefinite clinical significance and postnatal evolution, the parents continued with the pregnancy and took serial physical examinations (SPEs) for subsequent observations up to 5 months after the infant was born, which observed no delay in gross motor development. The patients appreciated the returned testing results and reported no regrets in having WES which was “the right decision” as commented by them in the follow‐up interview.

## 4 DISCUSSION

Right now, multiple evidence has supported that monogenic disorder is main causes of human CAKUT.^19^, ^20^ With the application of high‐throughput next‐generation sequencing, the clinical molecular genetic testing has been currently utilized in the diagnosis of monogenic causes in CAKUT patients. In a study by Amelie T et al, WES was performed to analyze the genotypes of 232 families, discovering that 13% of the families harbored a causative mutation in a known gene for isolated or syndromic CAKUT.^21^ In 2018, in 23.3% of the patients subjected to WES, Hila Milo identified candidate pathogenic variations in 625 genes which were related to Mendelian kidney and genitourinary disorders.^22^ Nevertheless, these studies mainly focus on postnatal cases, and the prenatal genetic diagnosis of CAKUT remains challenging due to genetic heterogeneity, phenotypic progressivity, and incomplete genetic penetrance. Hence, we used WES to investigate its application in the diagnosis of isolated CAKUT. Accordingly, we demonstrated 3 pathogenic and 1 likely pathogenic variants in three genes, *PKD1, ACTA2*, and *PKHD1*, which have been reported to be associated with clinical features consistent with bilateral renal anomalies (Table 2). Furthermore, the total diagnostic yield of our study was 7.3% (3/41). Thus, the detection rate was 0 for unilateral renal anomalies and 15.7% (3/19) for bilateral renal abnormalities, which is consistent with previous study reported by Maria showing 14.5% (9/62) of autopsied fetuses with isolated bilateral kidney anomalies carried likely deleterious variants.^23^ Obviously, the yield was higher when the fetuses were affected by bilateral renal abnormalities.

The value of prenatal WES in genetic diagnosis of CAKUT with/without other anomalies was thoroughly discussed in several recent studies (Table 3). Lei et al^24^ reported that 10% (3/30) of fetuses with unexplained CAKUT were diagnosed by WES. In 2019, Slavé Petrovski^8^ reported that 6.4%(15/234) of fetuses with structural anomalies were diagnosed with pathogenic mutations. The frequency of genetic diagnoses varied among different anatomical systems, such as lymphatic or effusion system (24%), skeletal system (24%), central nervous system (22%), and renal system (16%). Moreover, Jenny Lord et al^7^ performed the largest trio WES cohorts with unselected anomalies. Genetic variants were identified in 8.5% (52/610) of fetuses with structural anomalies while variants of uncertain significance with potential clinical utility were identified in an additional 3.9% (24/610) of fetuses. However, the diagnostic yield was 0 in the renal abnormalities group. In this study, we obtained monogenic diagnosis in 7.32% (3/41) of the fetuses with isolated CAKUT. Thus, the actual clinical diagnostic yield in our cohort was comparable with those in previous studies, although being slightly lower than some other studies.^8^, ^24^ One possible reason for the low detection rate could be attributed to small sample size in our study. Several genes can cause CAKUT. Up to now, more than 200 monogenic syndromes have been described involving renal or urinary anomalies as one of their features.^3^ Our research needs to expand the sample size. On the other hand, the varied proportion of individuals with isolated CAKUT was also related to the diagnostic rate. The detection rate in the study by Lei et al was 9.1% (2/22) for isolated CAKUT and 25% (2/8) for non‐isolated CAKUT.^24^ Additionally, the detection rate in the study by Slavé Petrovski was 8.3% (1/12) for isolated CAKUT and 23% (3/13) for non‐isolated CAKUT.^8^ However, Jenny Lord reported detected rate of 0 in renal abnormalities without other structural anomalies, and our cases were also all isolated renal abnormalities, which indicated that higher risk of monogenic defects in non‐isolated CAKUT than isolated CAKUT. Hence, monogenic mutation analysis should be particularly conducted if bilateral lesion and/or other extrarenal structural abnormalities were presented.

**Table 3:** Summary of the 5 studies included in genetic analysis of WES in fetuses with CAKUT

Study	Study design	Inclusion criteria for original study	Detect mode	Detection method	Sample size and detected rate		
					Total	Isolated CAKUT	Non‐ isolated CAKUT
Jenny 2019	Prospective study	Various fetal structural anomalies	Parent–fetus trios	WES	0% (0/16)	—	—
Slavé 2019	Prospective study	Various fetal structural anomalies	Parent–fetus trios	WES	16% (4/25)	—	—
Maria 2017	Prospective study	Autopsied Fetuses with prenatally diagnosed kidney anomalies	Parent–fetus trios	Gene panel and WES	14.5% (9/62)	14.5% (9/62)	Non
Lei 2017	Prospective study	Fetuses with CAKUT with/without other structural anomalies.	23 cases with only the proband/7 cases with parent–fetus trio	WES	13.3% (4/30)	9% (2/22)	25% (2/8)
This study	Prospective study	Fetuses with unexplained CAKUT without other structural anomalies	28 cases with parent–fetus trio/13 cases with only the proband	WES	7.3% (3/41)	7.3% (3/41)	Non

Abbreviations: CAKUT, congenital anomalies of the kidney and urinary tract; WES, whole‐exome sequencing.

Given the heterogeneous etiologies of hyperechoic enlarged kidneys, prenatal ultrasound cannot provide an accurate etiologic diagnosis for renal disorders, it is important to advice the family to identify the molecular etiology in prenatal diagnosis. Shirley Shuster evaluated 52 pregnancies with isolated bilateral hyperechogenic kidneys and found that 34 cases had enlarged kidneys, 16 cases had normal size kidneys, and 2 cases had small kidneys. Accordingly, 7 cases were diagnosed with variations in *PKD1* gene and 15 cases were affected by ARPKD.^25^ Furthermore, Leire Gondra reported that *HNF1B* mutation represents the leading cause of polyhydramnios associated with hyperechogenic (and sometimes enlarged) kidneys.^26^ In this study, a heterozygous missense mutation (*PKD1*: c.6571C > T) in a fetus with bilateral echogenic kidneys and two frame shift deletions (PKHD1:c.8301del and c.4481del) in a fetus with bilateral hyperechogenic and enlarged kidneys combined with oligohydramnios were identified. *PKD1* and PKHD1 are known to be associated with ADPKD and ARPKD. In a cohort of isolated bilateral hyperechogenic kidneys, Shirley Shuster et al found 7 AD‐PKD cases, among which 6 was inherited and 1 was found of carrying de novo causative *PKD1* variants.^25^ ARPDK, as commented by Adeva et al (2006),^27^ was usually considered as a typical infantile disorder with the enlarged echogenic kidneys detected in utero or neonatal period, resulting in neonatal death in most cases. Although echogenic kidneys without cysts are common in ultrasonographic findings, it potentially indicate the presence of significant renal disease.^28^

*ACTA2*, which encodes the alpha‐smooth muscle actin(α‐SMA), is mutated in MSMDS patients, which was first reported in a published work by Milewicz et al in 2010.^29^ In 2019, Sai‐Nan Chen et al^30^ found that all reported MSMDS patients worldwide were tested positive for the *ACTA2* gene mutations, and 81.25% of the mutations found was p.R179H. Apart from cardiovascular and cerebrovascular diseases, MSMDS patients also suffered from other organ dysfunctions including depressed systolic performance of the bladder and gastrointestinal tract, thus resulting in hypotonic bladder, hydronephrosis, hypospadias, intestinal malrotation, hypoperistalsis, and gallstones. However, here, we reported a prenatal case characterized by ultrasound abnormalities including oligohydramnion, bilateral echogenic kidneys, bilateral hydronephrosis, megalo‐ureter, and megabladder, and this distinct case presented a novel prenatal form of variants in *ACTA2* (p.R179H). Therefore, the above prenatal finding broadened the spectrum of *ACTA2* phenotypes.

In our study, we reported an incidental finding (*PPM1D:* c.1434delC) in a fetus with right renal dysplasia, which made a counseling challenges and deep ethical questions in the prenatal setting, including the fact that the return of any incidental finding (IF) may cause parental anxiety, even a pregnancy termination decision‐making. Currently, there is no official guided recommendation on how to report and counsel IF in prenatal setting. According to the ACMG guidelines, patients are advised to be informed of known and likely pathogenic variants in 59 disease‐specific genes regardless of indication for postnatal WES.^31^ However, it is controversial that the ACMG guidelines exclude prenatal WES, which has been discussed in some publications. Sarah Harris agreed that patients must be informed of the possibility of these results in advance. For variants located in genes which were reported to cause moderate to severe childhood onset disorders but not found to be related to phenotypes in fetus.^9^ Kristin G recommended that these variants should be included in the returned results if found to be highly penetrant pathogenic. Since many fetal disorders, including nonsyndromic intellectual disability, neurodevelopmental disorders, and metabolic disorder, cannot be diagnosed by fetal imaging, adequate pretest and post‐test counseling are therefore supplemented as a commonly acknowledged practice in this area.^12^ Experts also suggest performing WES on those highly selected cohorts when a specific syndrome is suspected.

## 5 CONCLUSION

Based on our current findings and previous reports, WES offers a supplementary approach for prenatal genetic diagnosis of isolated CAKUT with negative CMA result. The prenatal WES is more suitable for the etiologic diagnosis of diseases with higher risk of monogenic causes, such as bilateral renal malformation. The supplementary approach may benefit genetic counseling, decision‐making on pregnancy outcome, and future family planning.
